# Herbicidal properties of antihypertensive drugs: calcium channel blockers

**DOI:** 10.1038/s41598-021-93662-2

**Published:** 2021-07-09

**Authors:** Hannan Safiyyah Tan Sian Hui Abdullah, Poh Wai Chia, Dzolkhifli Omar, Tse Seng Chuah

**Affiliations:** 1 Faculty of Science and Marine Environment, Universiti Malaysia Terengganu, Kuala Nerus, Terengganu Malaysia; 2 Faculty of Agriculture, Universiti Putra Malaysia, Serdang, Selangor Malaysia; 3 Faculty of Plantation and Agrotechnology, Universiti Teknologi MARA, Arau, Perlis Malaysia

**Keywords:** Toxicology, Environmental impact

## Abstract

Herbicide resistance is a worldwide problem in weed control. This prompts researchers to look for new modes of action to slow down the evolution of herbicide-resistant weeds. This research aims to determine the herbicidal action of thiazolo[3,2-a]pyrimidines derivatives, which are well known as antihypertensive drugs. The phytotoxic effects of ten compounds were investigated using leaf disc discoloration test and seed germination bioassay. At concentrations of 125 to 250 mg/L, the 5-(3-Fluoro-phenyl)-7-methyl-5H-thiazolo[3,2-a]pyrimidine-6-carboxylic acid ethyl ester (**c**) was highly active against *Oldenlandia verticillata* and *Eleusine indica*. At application rates of 1.25 to 2.5 kg ai/ha, formulated **c** demonstrated selective post-emergence and pre-emergence herbicidal activity against *O. verticillata*, *E. indica* and *Cyperus iria*. In the crop tolerance test, formulated **c** outperformed the commercial herbicide diuron, with aerobic *Oryza sativa* being the most tolerant, followed by *Zea mays*, and *Brassica rapa*. The addition of calcium chloride partially nullified compound **c**'s inhibitory effects on weed shoot growth, indicating that it has potential as a calcium channel blocker. Compound **c** acted by triggering electrolyte leakage without affecting photosystem II. These findings imply that **c** could be explored further as a template for developing new herbicides with novel modes of action.

## Introduction

Over the past, some pharmaceuticals, which have been credited for saving millions of lives, were found to possess the property of pesticide^1,2^. Both pesticides and drugs are following similar regulations in terms of design and production^3,4^. A well-known example is fluconazole, a pharmaceutical fungicide product that is now patented as an agricultural product^1^. The endothall herbicide, which is effective against aquatic and terrestrial plants, is now listed as a chemical scaffold for developing new antimalaria lead^5^.

Calcium channel blockers are important drug targets among pharmacologists due to their ultimate role in treating hypertension^6,7^. Basically, the calcium (Ca^2+^) channel family can be subdivided into three categories: L-type (Ca_v_1), P/Q, N and R-types (Ca_v_2) and T-type (Ca_v_3)^8^. Along these lines, numerous studies have revealed that different subtypes of calcium channels are associated with cardiovascular^6,9^ and neuropsychiatric^7,10^ disorders and cancer^8,11^. At a therapeutic dose, the calcium channel blockers decrease the elevated blood pressure of hypertensive patients. However, they do not alter the blood pressure of normotensive individuals as was found in animals. Several major families of calcium channel blockers are verapamil, nifedipine, diltiazem, cinnarizine, bepridil and mibefradil^12^.

In plant stress adaptation and growth processes, calcium ion (Ca^2+^) has been identified to constitute the key transducer and regulator^13^. Hence, the presence of calcium channel blockers could inhibit the influx and increase the efflux of calcium ions from plant cells^14,15^. Studies have shown that verapamil inhibits the influx of Ca^2+^ into the root apex of rice and wheat, while nifedipine prevents the development of root hair and pollen grain germination of Arabidopsis^16^. The calcium antagonist properties are displayed by a broad number of pure natural compounds, primarily coumarins, which have been identified and isolated from plants^14,17^.

Thiazolopyrimidine, an example of calcium channel blocker, has been of interest due to their well-known biological and medicinal activities^18–25^. Despite their wide range of pharmacological activities, there is a lack of information regarding the phytotoxic behaviour of these fused pyrimidine derivatives. For the past 30 years, there are no novel herbicides with new sites of action being introduced into the market, making growers highly dependent on existing herbicides, thereby leading to evolution of herbicide resistance in weed, which indirectly imposed new challenges for weed management. Hence, this study aimed to screen for the promising thiazolopyrimidine derivatives and evaluate the pre-and post-herbicidal activity of selected thiazolopyrimidine derivative in several weeds and crop species. The effects of the selected thiazolopyrimidine derivative on quantum yield and ion leakage were carried out to reveal its potential mode of action, while the role of metal chloride to antagonize herbicidal action of the selected thiazolopyrimidine was also determined.

## Results

### Leaf disc discolouration test

**Figure 1:**
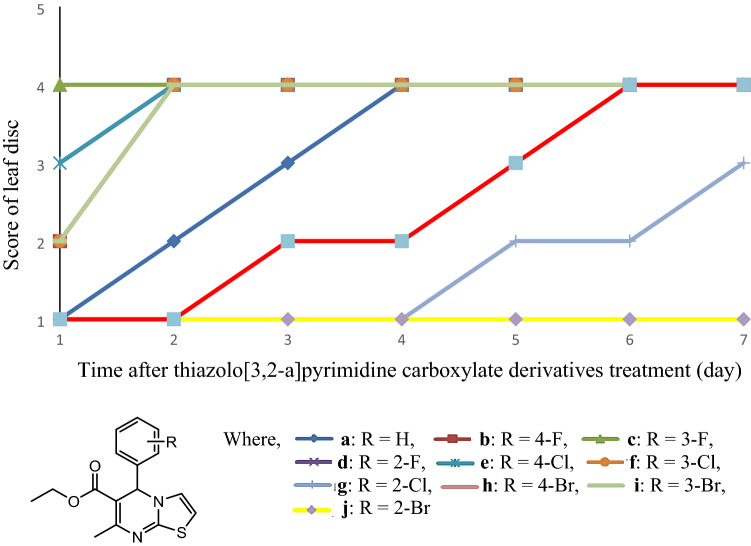
Phytotoxic effect of thiazolo[3,2-a]pyrimidine carboxylate derivatives at 500 mg/L on green pigment degradation of *Oldenlandia verticillata* throughout seven days of incubation period. Each score representing different colour retention of leaf disc. Score 1: Green—The leaf disc surface is completely green; Score 2: Green brownish—10–30% of leaf disc surface is dark brown in colour; Score 3: Brown greenish—50–80% of leaf disc surface is dark brown in colour; Score 4: Dark brown—More than 90% of leaf disc surface, in aggregate, is dark brown.

**Figure 2:**
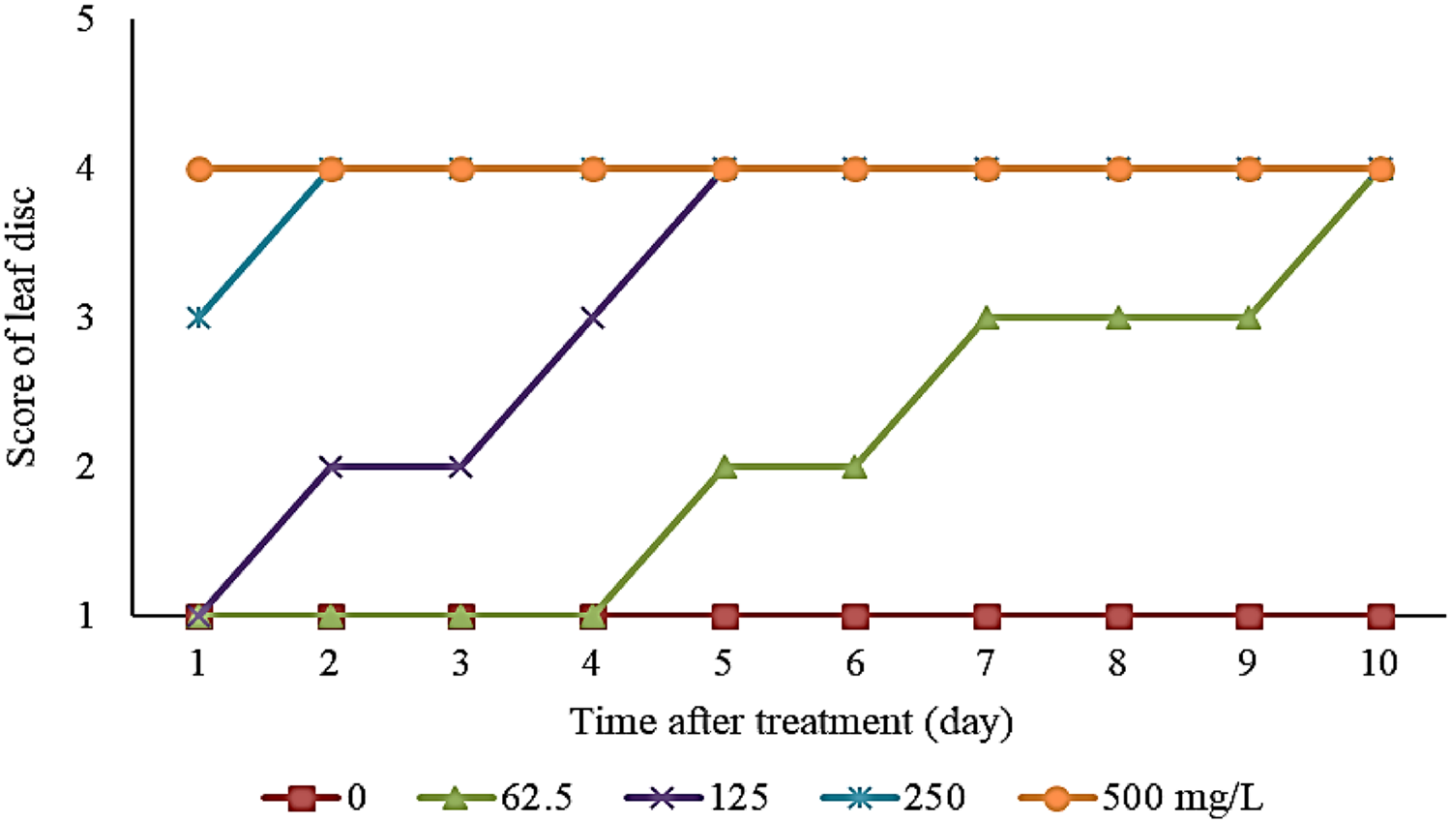
Phytotoxic effects of 5-(3-Fluoro-phenyl)-7-methyl-5H-thiazolo[3,2-a]pyrimidine-6-carboxylic acid ethyl ester (**c**) at 0, 62.5,125, 250 and 500 mg/L on green pigment degradation of *Oldenlandia verticillata* throughout 10 days of incubation period. Each score representing different colour retention of leaf disc. Score 1: Green—The leaf disc surface is completely green; Score 2: Green brownish—10–30% of leaf disc surface is dark brown in colour; Score 3: Brown greenish—50–80% of leaf disc surface is dark brown in colour; Score 4: Dark brown—More than 90% of the leaf disc surface, in aggregate, is dark brown.

Results have shown that compound **c** at a concentration of 500 mg/L exhibited the most phytotoxic effect in which it displayed a score of 4 even on day 1 of treatment followed by compound **d**, **e**, **b**, **f**, **h** and **i** on day 2, and lastly compound **a** on day 4 of treatment (Fig. 1). The complete diminish in the green colour of the diuron-exerted *Oldenlandia verticillata* leaf was recorded on day 6. Of the ten compounds, however, compound **g** and **j** did not achieve a score of 4 within 7 days of treatment which implies they are less phytotoxic compared to other compounds. Compound **c** was further undergone leaf disc discolouration test at concentrations of 0, 62.5, 125, 250 and 500 mg/L (Fig. 2), and apparently, 125 mg/L is the lowest concentration for it to exhibit the full phytotoxicity within 7 days of incubation.

### Germination test

**Figure 3:**
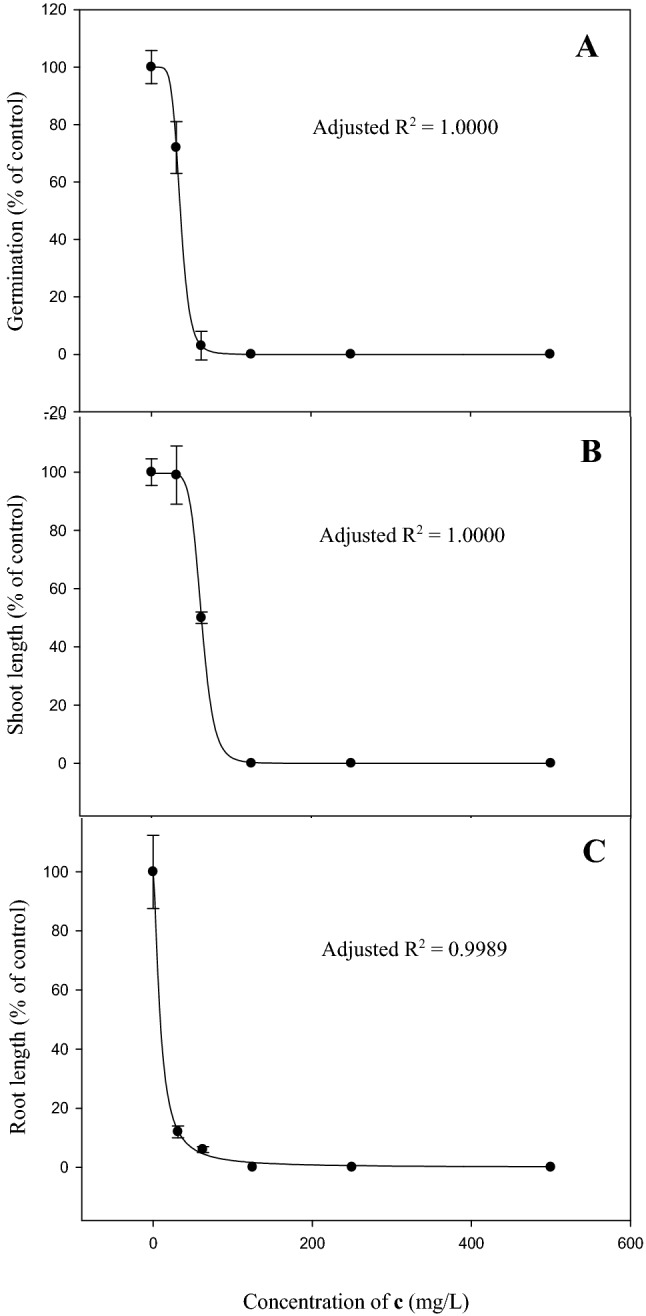
Inhibitory effect of 5-(3-Fluoro-phenyl)-7-methyl-5H-thiazolo[3,2-a]pyrimidine-6-carboxylic acid ethyl ester (**c**) on germination (**A**), shoot length (**B**) and root length (**C**) of *Eleusine indica* after 7 days of treatment. The standard deviation of the mean is symbolized by the vertical bar.

At 500 mg/L concentration, compound **b**, **c**, **e**, **f**, **g**, **j** and diuron provided complete inhibition of *Eleusine indica* germination and root growth (Table 1). Visibly, all compounds suggested a lower reduction of shoot length than that of the root, where the shoot lengths were more than 50% of control except for compound **c,** in which seed germination and seedling growth were inhibited entirely. Figure 3 shows the inhibition on seed germination, shoot and root growth of bioassay species when compound **c** applied at concentrations of 62.5, 125, 250, and 500 mg/L. This effect was most noticeable with an increasing concentration of **c**, where full inhibition was observed at a concentration of as low as 125 mg/L. On the contrary, the shoot growth of *E. indica* was less suppressed at lower concentrations of 31.25 and 62.5 mg/L. The minimum concentration for compound **c** to show completion inhibition is 125 mg/L. This corresponds to the leaf disc test result, which showed a complete diminish in green colour at the similar concentration on day 5. The concentration of compound **c,** which gives 50% inhibition (ED_50_) on *E. indica* was described in Table 2, showing the most inhibitory effect on root growth (9 mg/L), followed by germination (39 mg/L) and shoot growth (63 mg/L).

**Table 1:** Inhibitory effect (mean ± standard error) of thiazolo[3,2-a]pyrimidine carboxylate derivatives at 500 mg/L on germination, shoot length and root length of *Eleusine indica* after 7 days. of treatment


Compound	R	Average percentage of control (%)		
		Germination	Shoot length	Root length
**a**	H	10 ± 0	70 ± 9.0	13 ± 3.1
**b**	4-F	0 ± 0	78 ± 3.9	0 ± 0
**c**	3-F	0 ± 0	0 ± 0	0 ± 0
**d**	2-F	53 ± 15.0	92 ± 7.5	14 ± 1.4
**e**	4-Cl	0 ± 0	64 ± 4.8	0 ± 0
**f**	3-Cl	0 ± 0	50 ± 10.3	0 ± 0
**g**	2-Cl	0 ± 0	104 ± 7.3	0 ± 0
**h**	4-Br	20 ± 0	64 ± 6.9	11 ± 0
**i**	3-Br	10 ± 0	81 ± 0	11 ± 0
**j**	2-Br	0 ± 0	72 ± 17.7	0 ± 0
diuron		0 ± 0	0 ± 0	0 ± 0

**a**: 7-Methyl-5-phenyl-5H-thiazolo[3,2-a]pyrimidine-6-carboxylic acid ethyl ester; **b**: 5-(4-Fluoro-phenyl)-7-methyl-5H-thiazolo[3,2-a]pyrimidine-6-carboxylic acid ethyl ester; **c**: 5-(3-Fluoro-phenyl)-7-methyl-5H-thiazolo[3,2-a]pyrimidine-6-carboxylic acid ethyl ester; **d**: 5-(2-Fluoro-phenyl)-7-methyl-5H-thiazolo[3,2-a]pyrimidine-6-carboxylic acid ethyl ester; **e**: 5-(4-Chloro-phenyl)-7-methyl-5H-thiazolo[3,2-a]pyrimidine-6-carboxylic acid ethyl ester; **f**: 5-(3-Chloro-phenyl)-7-methyl-5H-thiazolo[3,2-a]pyrimidine-6-carboxylic acid ethyl ester; **g**: 5-(2-Chloro-phenyl)-7-methyl-5H-thiazolo[3,2-a]pyrimidine-6-carboxylic acid ethyl ester; **h**: 5-(4-Bromo-phenyl)-7-methyl-5H-thiazolo[3,2-a]pyrimidine-6-carboxylic acid ethyl ester; **i**: 5-(3-Bromo-phenyl)-7-methyl-5H-thiazolo[3,2-a]pyrimidine-6-carboxylic acid ethyl ester; **j**: 5-(2-Bromo-phenyl)-7-methyl-5H-thiazolo[3,2-a]pyrimidine-6-carboxylic acid ethyl ester.

**Table 2:** ED_50_ values of compound **c** for *Eleusine indica* after 7 days of treatment with germination test. ED_50_ (mean ± standard error) is the dose needed by the compound to reduce germination, shoot length and root length by 50%.

Compound	ED_50_ (mg/L)		
	Germination	Shoot length	Root length
c	36 ± 0.1	63 ± 0.1	9 ± 3

### Post-emergence application

**Figure 4:**
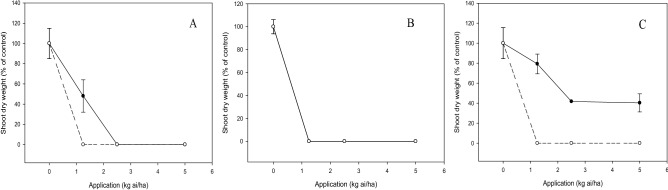
Herbicidal effects of **c** (solid line) and diuron (dashed line) treatments on shoot dry weight of *Eleusine indica* (**A**), *Oldenlandia verticillata* (**B**) and *Cyperus iria* (**C**) respectively 3 weeks after post-emergence application.

Significant interaction (*p* < 0.05) between application rate and tested compound on shoot growth of bioassay species was noted depending on types of test weeds and crops. Inhibitory effects of compound **c** on *E. indica* and *C. iria*. were lower than those of diuron when increasing the application rate. At 1.25 kg ai/ha, compound **c** reduced the shoot dry weight of *E. indica* and *C. iria* by 50% and 20%, respectively, whereas diuron gave complete inhibition of the weeds (Fig. 4). However, the shoot dry weight of *O. verticillata* was entirely inhibited by compound **c** and diuron at 1.25 kg ai/ha. These results imply that the degree of herbicidal activity of compound **c** on the test weeds can be classified in order of decreasing inhibition as follows: *O. verticillata*, *E. indica* and *C. ria* (Fig. 4).

**Figure 5:**
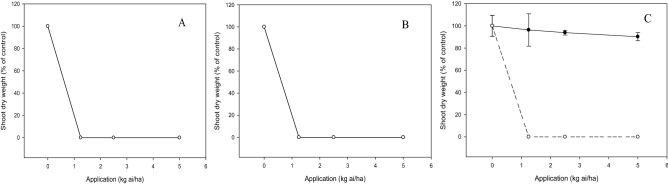
Phytotoxic effects of **c** (solid line) and diuron (dashed line) treatments on shoot dry weight of *Brassica rapa* (**A**), *Zea mays* (**B**) and *Oryza sativa* (**C**) respectively 3 weeks after post-emergence application.

For crops, both **c** and diuron exhibited substantial phytotoxic activity by exerting complete suppression on the shoot growth of *B. rapa* and *Z. mays* at the lowest application rate of 1.25 kg a/ha. By contrast, aerobic *O. sativa* was found to be tolerant to compound **c** even at the highest application rate of 5 kg ai/ha, while diuron inhibited shoot growth of the crop completely at 1.25 kg ai/ha (Fig. 5).

### Pre-emergence application

**Figure 6:**
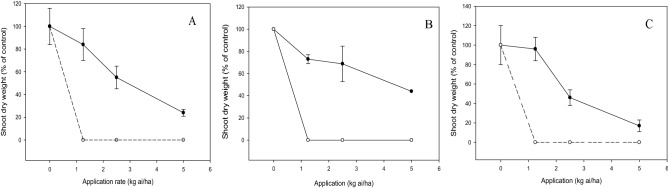
Herbicidal effects of **c** (solid line) and diuron (dashed line) treatments on shoot dry weight of *Eleusine indica* (**A**), *Oldenlandia verticillata* (**B**), and *Cyperus iria* (**C**) respectively 2 weeks after pre-emergence application.

**Figure 7:**
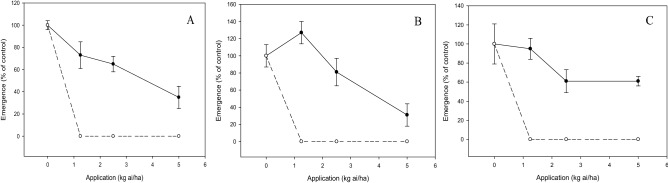
Herbicidal effects of **c** (solid line) and diuron (dashed line) treatments on seedling emergence of *Eleusine indica* (**A**), *Oldenlandia verticillata* (**B**), and *Cyperus iria* (**C**) respectively 2 weeks after pre-emergence application.

**Figure 8:**
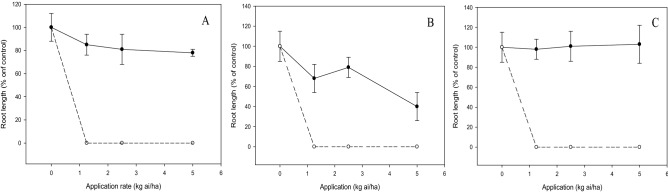
Herbicidal effects of **c** (solid line) and diuron (dashed line) treatments on root length of *Eleusine indica* (**A**), *Oldenlandia verticillata* (**B**), and *Cyperus iria* (**C**) respectively 2 weeks after pre-emergence application.

There was a significant interaction (*p* < 0.05) between the tested compound and application rate on shoot growth, emergence and root growth of test weeds and crops. The increased dose of compound **c** and diuron decreased the shoot dry weight of weeds. At 2.5 kg ai/ha, compound **c** inhibited the weeds by 40 to 60%, whereas diuron provided complete weed inhibition at a similar application rate (Fig. 6). Likewise, weed seedling emergence was reduced by diuron and compound **c** in a dose-dependent manner, but more significant herbicidal activity was exhibited by diuron than compound **c** (Fig. 7). Application of compound **c** at 2.5 kg ai/ha was able to suppress 20 to 40% weed emergence, whereas complete inhibition was evident for diuron at a similar dose. On the other hand, compound **c** had less inhibition on root growth of weeds, with *C. iria* being the least susceptible, followed by *E. indica* and *O. verticillata* when increasing the dose. By contrast, complete inhibition of weed root growth was observed at 1.25 kg ai/ha diuron (Fig. 8).

**Figure 9:**
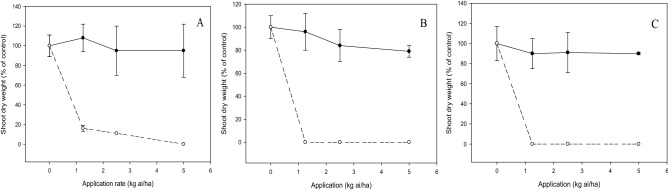
Phytotoxic effects of **c** (solid line) and diuron (dashed line) treatment on shoot dry weight of *Brassica rapa* (**A**), *Oryza sativa* (**B**) and *Zea mays* (**C**) 2 weeks after pre-emergence application.

**Figure 10:**
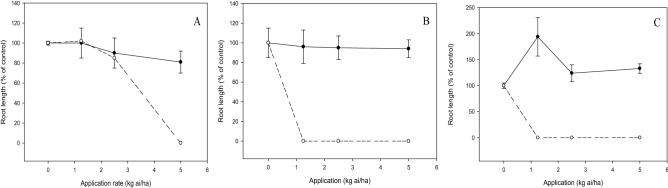
Phytotoxic effects of **c** (soild line) and diuron (dashed line) treatment on root length of *Brassica rapa* (**A**), *Oryza sativa* (**B**) and *Zea mays* (**C**) 2 weeks after pre-emergence application.

The shoot growths of test crops were not significantly (*p* > 0.05) affected by compound **c** with increasing dose even at the maximum application rate of 5.0 kg ai/ha (Fig. 9). Similarly, the increased dose of compound **c** did not inhibit the root growths of the crops significantly (*p* > 0.05), with *Z. mays* being the most tolerant, followed by aerobic *O. sativa* and *B. rapa* (Fig. 10). On the contrary, diuron demonstrated excellent inhibitory property by reducing the shoot dry weight (Fig. 9) and root length (Fig. 10) of the crops by 100%, even at the lowest application rate of 1.25 kg ai/ha.

### Electrolyte leakage determination

In this study, diuron did not cause significant electrolyte leakage in *O. verticillata* at concentrations ranging from 100 to 300 mg/L during the 24-h incubation period (Table 3). At concentration 100 mg/L, electrical leakage of *O. verticillata* was not notable when treated with compound **c**. However, the electrolyte leakage was enhanced when the concentration of **c** increased from 200 to 300 mg/L.

**Table 3:** Electrolyte leakage of *Oldenlandia verticillata* leaf disc after treatments of compound **c** and diuron. Data show the mean ± standard deviation of six replicates.

Treatment (mg/L)	Average percentage of control (%)					
	Compound **c**			Diuron		
	3 H	18 H	24 H	3 H	18 H	24 H
100	99 ± 6.2^a^	116 ± 0.5^a^	119 ± 15.8^a^	77 ± 9.1^a^	90 ± 13.1^a^	96 ± 2.9^a^
200	131 ± 7.3^a^	156 ± 8.6^b^	166 ± 4.2^b^	103 ± 10.0^a^	118 ± 23.4^a^	123 ± 25.9^a^
300	225 ± 11.1^a^	302 ± 4.2^b^	362 ± 6.7^c^	89 ± 22.5^a^	124 ± 19.4^a^	114 ± 12.2^a^

*Means followed by similar small letters within the same row have no significant differences (*p* > 0.05) among incubation periods within compound **c** or diuron.

### Quantum yield determination

Treatment with diuron throughout all the concentrations indicated zero to low level of photosynthetic electron flow with no significant difference when *O. verticillata* was incubated for 24 h. However, this is not the case for compound **c**, where it did not exert any effects on quantum yield (Table 4).

**Table 4:** Quantum yields of leaf discs of *Oldenlandia verticillata* after treatments of compound **c** and diuron. Data indicate the mean ± standard deviation of six replicates.

Treatment (mg/L)	Percentage of control (%)					
	s			Diuron		
	3 H	18 H	24 H	3 H	18 H	24 H
100	101 ± 4.2^a^	97 ± 8.1^a^	102 ± 5.9^a^	0 ± 0	0 ± 0	0 ± 0
200	102 ± 9.1^a^	103 ± 13.5^a^	104 ± 10.5^a^	0 ± 0	0 ± 0	0 ± 0
300	102 ± 5.9^a^	108 ± 10.5^a^	103 ± 3.5^a^	0.4 ± 0.6^a^	0.6 ± 0.6^a^	1.2 ± 1.9^a^

*Means followed by similar small letters within the same row have no significant differences (*p* > 0.05) among incubation periods within compound **c** or diuron.

### Calcium channel antagonist determination

As shown in Table 5, compound **c** at 125 mg/L indicated higher inhibitory effects than diuron either on *E. indica* seed germination, shoot or root growths. Moreover, at the similar concentration, the treatment of diuron solely and the combination of metal chloride solutions with diuron portraited a lower inhibitory effect than compound **c**. With the supplementation of Ca^2+^ to **c**, only the shoot length of *E. indica* was recovered.

**Table 5:** Inhibitory effect of **c** (125 mg/L) and diuron (125 mg/L) added with or without metal chlorides (50 mg/L) on germination, shoot growth and root growth of *Eleusine indica* after 7 days of treatment. Data indicate the mean ± standard deviation of five replicates.

Treatment	Germination	Shoot length	Root length
		(% of control)	
**c**	0^a^	0^a^	0^a^
diuron	83 ± 6^b^	75 ± 13^c^	19 ± 1^b^
CaCl_2_	94 ± 7^bc^	99 ± 6^de^	106 ± 15^c^
KCl	96 ± 8^bc^	108 ± 8^e^	104 ± 14^c^
NaCl	102 ± 4^c^	107 ± 7^e^	107 ± 9^c^
**c** + CaCl_2_	0^a^	30 ± 2^b^	0^a^
**c** + KCl	0^a^	0^a^	0^a^
**c** + NaCl	0^a^	0^a^	0^a^
diuron + CaCl_2_	94 ± 6^bc^	77 ± 6^c^	21 ± 2^b^
diuron + KCl	94 ± 6^bc^	86 ± 13^cd^	25 ± 6^b^
diuron + NaCl	91 ± 13^bc^	88 ± 12^cd^	22 ± 3^b^

*Means followed by similar small letters within the same column have no significant differences (*p* > 0.05) among treatments.

## Discussion

Compared with chlorine and bromine derivatives, compounds containing fluorine atom demonstrated a faster colour change on leaf discs, such as **c**, **b** and **d** (Fig. 1). This is evident as many commerical synthetic herbicides, such as dithiopyr, ethoxyten, bitafenacil, metflurazon and fluridone were found to contain at least one or more fluorinated atoms within the molecules^26^.

As seeds grow, they not only depend on the roots for water and nutrient uptake but also it is their shoots that perform photosynthesis which enables the seedling to grow healthier. In this in vitro germination experiment, compounds were added to the filter papers in the Petri dish, where the seedling roots develop. Thus, it is reasonable that compound **c** exerted greater inhibition on the roots than shoots (Table 1), as shown by the study of Bourgou et al*.*^27^, where greater inhibitory effects of the phenolic compounds were observed on roots if compared to shoots.

The outcomes from germination bioassays implied that compound **c** is a strong root and germination inhibitor. It is the most phytotoxic among all the derivatives of thiazolopyrimidine, and this finding is consistent with previously performed leaf disc assays. Research done by Abe and Kameya^28^ discovered that 430 μM (approximately 100.23 mg/L) of diuron inhibited asparagus seed germination by 65%. Unlike asparagus, complete inhibition of *E. indica* germination by diuron was found at 500 mg/L.

During post-emergence application on the weeds, generally, the inhibitory effect of **c** on shoot growth was varied with application levels, with increased suppression at higher rates. In our study, the optimum rate of **c** to fully suppress the shoot growth of *E. indica* was 2.5 kg ai/ha (Fig. 4) whilst the early post-emergence study of rice herbicide, propanil combined with thiobencarb at 3.6 kg ai/ha conducted by Norhafizah et al.^29^ was found to decrease the shoot fresh weight of *E. indica* greater than 90%. Although diuron fully inhibited the shoot growth of *O. verticillata* at the beginning first week of all treatments, the seedlings treated by compound **c** was still able to grow (data not shown). However, they gradually wilted and died after 3 weeks of treatment. These findings indicate that when introduced to *O. verticillata* seedlings, the phytotoxicity of **c** is comparable to that of diuron (Fig. 4).

A good selective herbicide helps with weed control but does not harm the crops at the same time. In this research, compound **c** is not suitable to be applied as a post-emergence herbicide in *B. rapa* and *Z. mays* since complete inhibition of crop growth is evident irrespective of any application rate. Nonetheless, the treatment on aerobic *O. sativa* yielded promising results because no significant effect was observed when treated with **c** at even 5.0 kg ai/ha (Fig. 5). In an experiment by Mahmoudi et al*.*^30^, the application of thiobencarb at the rate of 3.16 kg ai/ha resulted in minor rice injury, and they proposed that 3.16 kg ai/ha thiobencarb should be applied in Iranian paddy fields. This rate is slightly higher than the effective rate of **c** (2.5 ka ai/ha) for weed control, suggesting that compound **c** is a potential alternative of thiobencarb for weed control in *O. sativa*.

Post-emergence herbicide usually requires a higher application rate than pre-emergence herbicide as the latter is applied to kill weeds before emerging from the soil. Nonetheless, the findings from this research indicated that post-emergence treatment has an overall lower effective application rate than pre-emergence treatment. These can be explained by the fact that soil pH determines the adsorption and desorption of herbicide in soil which in turn can affect its bioavailability to the target plant^31,32^. In this study, being a weak acid compound such as **c**, the low soil pH of 4.7 may have increased the binding of compound **c** to soil particle, thus leading to lower herbicidal activity when applied as pre-emergence control.

Time-course studies measuring the electrical conductivity of the bathing medium during an initial 18-h dark incubation followed by 6-h light exposure may distinguish compounds with the light-dependent mode of action from light-independent compounds^33^. In our study, diuron suggested a negligible ions leakage (Table 3). This result is consistent with that reported by Dayan and Watson^33^, who demonstrated that no significant electrolyte leakage of cotyledon cucumber discs was observed when examined PSII inhibitors such as diuron, atrazine and bentazon. In contrast to diuron, **c** was able to break down the membrane integrity since the ion leakage degree was parallel to the time increase at a low concentration of 200 mg/L. Likewise, the percentage of leakage by leaves of *Vicia faba* plants treated with calcium channel blockers of verapamil and nifedipine were greater than those of non-treated plants^15^. At a higher concentration of 300 mg/L, the properties of the light-dependent mechanism of action for **c** are more apparent.

Low quantum yield is correlated with inhibition of photosynthesis. Diuron is known to interact directly with the photosynthetic electron flux, resulting in a strong chlorophyll fluorescence^34^. This can be seen when *O. verticillata* was incubated for 24 h and treated with diuron at 100, 200 and 300 mg/L; it showed zero to very low level of electron flow (Table 4). In other words, diuron has contributed to a rapid reduction of quantum yields irrespective of any concentration and incubation period. This is in line with the outcome of Dayan and Zaccaro^34^, in which diuron acted drastically, blocking nearly 100% of electron flow within 3 h of dark incubation and retaining this degree of inhibition throughout the experiment when cucumber cotyledon discs were exposed to diuron at 100 μM. On the contrary, compound **c** showed a negligible effect on the photosynthetic electron transport system. It did not induce any inhibition of electron flow during the whole incubation cycle, suggesting that **c** does not target the electron transport system of the plant.

The key concern with calcium ion (Ca^2+^), potassium ion (K^+^) and sodium ion (Na^+^) selection is attributed to their essential roles and transport for optimum plant growth^35^. At the onset of this study, no example of the thiazolo[3,2-a]pyrimidine derivatives was reported as calcium channel blocker in the plant. We were, thus, intrigued by the possibility of thiazolopyrimidine compound as plants’ calcium channel antagonist despite its well-known properties in animals^20^.

Since diuron is not a calcium channel blocker, adding calcium ions does not affect the diuron’s inhibitory effect (Table 5). At 125 mg/L, the phytotoxic effect of **c** was higher than that of diuron. This explained why the treatment of diuron or mixture of diuron and metal chloride solutions exerted a lower inhibitory effect than that of **c** at a similar concentration. Although inhibition of both shoot and root growths occurred before the supplementation of CaCl_2_ to compound **c**, the shoot was partially recovered after the addition of calcium salt. This claim can be supported by the study of Cho and Hong^36^ where calcium channel blockers decreased inhibition of the auxin-induced shoot growth of sunflower by supplying Ca^2+^. Abdel-Basset^15^ reported that efflux of Ca^2+^ given by the first leaf of *V. faba* plant irrigated with CaCl_2_ and subjected to verapamil and nifedipine treatments did not differ significantly with those of non-treated plant irrigated with CaCl_2_. These findings imply that the supplement of Ca^2+^ could ameliorate the adverse impact of calcium channel blockers.

In this research, KCl and NaCl served as a comparison to demonstrate the antagonistic effect of CaCl_2_ at 50 mg/L, and it was found that only the addition of Ca^2+^ can improve the elongation of shoot. Similar to the present findings, Cho and Hong^36^ documented that a low concentration of KCl and NaCl at 0.05 mM failed to antagonize the inhibitory effect of verapamil on shoot elongation of the auxin-induced sunflower. By contrast, CaCl_2_ at 0.05 mM antagonized the effect of verapamil effectively. The calcium channel antagonist property of **c** was identical to that represented by verapamil, suggesting that **c** is a calcium channel blocker.

## Conclusions

Thiazolo[3,2-a]pyrimidine carboxylate derivatives showed varying degrees of inhibitory effects on seed germination, shoot and root growth of *E. indica* in addition to leaf disc discolouration of *O. verticillata*. Of the ten synthesized compounds, compound **c** (5-(3-Fluoro-phenyl)-7-methyl-5H-thiazolo[3,2-a]pyrimidine-6-carboxylic acid ethyl ester) exhibited excellent herbicide properties at a low concentration of 125 mg/L where it fully inhibited the seedling growth and germination of bioassay species within 7 days after treatment. Although compound **c** did not exhibit strong inhibitory activity when applied as post-and pre-emergence herbicides, as shown by diuron in this study, the crop species were tolerant to **c** without decreasing its herbicidal efficacy to the weed species. Compound **c** is a better choice than diuron when applied as a pre-emergence herbicide for weed control in *Z. mays*, *B. rapa* and aerobic *O. sativa*. Meanwhile, compound **c** is suitable for post-emergence treatment to provide weed control in aerobic *O. sativa*. Moreover, compound **c** demonstrated calcium channel blocking activity and promoted electrolyte leakage, but it was not a photosystem II inhibitor.

Such discoveries are important because they describe new active ingredients and novel herbicide mode of action, thus providing another choice to increase the variety of available herbicides to slow down the evolution of herbicide resistance nowadays. Since compound **c** can be easily synthesized from one-pot multicomponent reaction^25^ of which the reactants are readily available on the market, it is rational and economically feasible to produce this compound on a commercial scale as a future herbicide for sustainable weed management. More in-depth research on the volume, concentration and ratio of the formulation that could increase the efficacy of compound **c** is desirable. Future challenges include an understanding of the persistence of this compound in the environment are of great importance. More research should also be conducted to elucidate this compound's mechanism action as a calcium channel blocker.

## Materials and methods

The following experiments on plants were performed in accordance with relevant guidelines and regulations and had been approved by Universiti Malaysia Terengganu (UMT), Malaysia.

### Bioassay species

A total of three weed species, namely *Eleusine indica* (grass), *Oldenlandia verticillata* (broadleaf) and *Cyperus iria* (sedge) and three crops, *Oryza sativa* (paddy), *Zea mays* (corn) and *Brassica rapa* (mustard), were used as bioassays. These weed species were selected as bioassay species due to their invasiveness and high abundance in aerobic rice fields, oil palm plantations, corn and vegetable farms. All the weed species were collected from UMT campus at Bukit Kor, Marang, Terengganu (5º12ʼN, 103º12ʼE). *Z. mays* and *B. rapa* seeds were purchased from Soon Huat Seeds Co. Sdn. Bhd. and Chiap Hup Agriculture Development Sdn. Bhd. respectively, whereas seeds of aerobic *O. sativa* were provided by the Malaysian Agricultural Research and Development Institute (Seberang Perai, Penang, Malaysia).

### Synthetic herbicide, thiazolo[3,2-a]pyrimidine-6-carboxylate derivatives and surfactants

The technical grade of diuron with 97% purity was provided by Ancom Corp Care Sdn. Bhd. Ten thiazolo[3,2-a]pyrimidine-6-carboxylate derivatives were synthesized via a one-pot multicomponent reaction of benzaldehyde derivatives, 2-aminothiazole and ethyl acetoacetate^37^ (Supplemental Figs. S1–S10). Surfactant alkylpolyglycoside (MBL 510H) and ethoxylated castor oil (Thermul 1284) were purchased from BASF and Huntsmann Corporation Australia Pty Ltd.

### Leaf disc discolouration test

Diuron and pure compounds of thiazolo[3,2-a]pyrimidine-6-carboxylate derivatives (Table 1) were respectively dissolved in ethyl acetate to produce 1 ml of solution at 500 mg/L and evaporated to dryness in 3-cm diameter Petri dishes under a fume hood. Then, 1 mL of distilled water was added to all compounds, and the Petri dishes were left overnight. Leaf discs of *O. verticillata* with 5 mm diameter were punched from fully developed leaves. One leaf disc was dipped into each Petri dish containing the solution in a growth chamber at 30/20 °C with a 12 h photoperiod. The photosynthetic photon flux density of the fluorescent lamps in the growth chamber was maintained at 100 μm m^−2^ s^−1^. Ethyl acetate was used as control negative and evaporated to dryness, and 1 mL of distilled water was added as described above. Leaf disc colour retention was scored daily for 7 days by referring to the modified method of Itoh et al*.*^38^ (Supplemental Table S1). Subsequently, the most phytotoxic compound of thiazolo[3,2-a]pyrimidine-6-carboxylate derivative was repeated with leaf disc bioassay at a concentration series 0, 62.5, 125, 250 and 500 mg/L throughout 10 days of the incubation period.

### Germination test

The seed coat scarification of *E. indica* was done with sandpaper and soaked in a 0.2% potassium nitrate solution overnight to break seed dormancy. Seed viability was tested to ensure seed germination is greater than 90%. Diuron and pure compounds of thiazolo[3,2-a]pyrimidine-6-carboxylate derivatives (Table 1) were respectively dissolved in ethyl acetate to produce 2 ml of solution at 500 mg/L in 5-cm diameter Petri dishes. Upon evaporation, another 2 mL of distilled water was added to each compound and left overnight. Ten scarified seeds of *E. indica* were then placed in the Petri dishes lined with two pieces of filter papers (Filtres Fioroni™) in the growth chamber. Ethyl acetate was used as control negative and evaporated to dryness as described above. Seedling growth, shoot length, and root length of *E. indica* was recorded after a week^39^. Seeds are considered to germinate when the root has protruded more than 2 mm, and data were expressed as a percentage of control. The most phytotoxic compound of thiazolo[3,2-a]pyrimidine-6-carboxylate derivative from germination bioassay was then selected and performed a 7-day germination test again at 0, 62.5, 125, 250 and 500 mg/L*.*

### Formulation of herbicide

#### Emulsion concentrate (EC) formulation

A 5% compound **c** or diuron post-emergence herbicide formulation was prepared by combining acetone dissolved compound **c** or diuron with surfactants, ethoxylated castor oil (20%) and alkylpolyglycoside (20%) and water (55%) based on the modified method of Lim et al*.*^40^.

#### Wettable powder (WP) formulation

Pre-emergence herbicide formulation was done using 5% compound **c** or diuron by blending acetone dissolved compound **c** or diuron with carriers, kaolinite (61%), silica oxide (25%) and gibbsite (9%) and drying based on the modified method of Norsworthy and Meehan^41^.

#### Post-emergence herbicidal test

Initially, 40 g of loam soil (pH 4.7, organic carbon 1.7%) was weighted into a plastic cup (5.8 × 7 cm). Next, the weeds and crop species that had reached the 2-leaf growth stage were transferred from seedling trays containing peat moss (Free Peat, Holland) at one plant per cup and was left a day for the plant to adapt to new soil condition. There were 5 replicates for each treatment with a plant per replicate of negative control, diuron and compound **c**, respectively. Three days after transplanting, the formulations prepared previously were sprayed directly onto the plants at 0, 1.25, 2.5, and 5.0 kg ai/ha, respectively, with a handheld sprayer. The treated plants were placed in the glasshouse. After 21 days of treatments, the plant tissues above the ground sections were harvested. Shoot dry weight data were recorded and expressed in percentage respective to control negative, which comprised the surfactants and water.

#### Pre-emergence herbicidal test

Formulated compound **c** and diuron were applied at rates of 0, 1.25, 2.5, and 5.0 kg ai/ha, respectively, into a plastic cup containing 40 g of loam soil. Non-treated cup (control negative) was applied with acetone combined with carriers of kaolinite, silica oxide and gibbsite and evaporated to dryness. Then, pre-germinated weed species (10 seeds per replicate) and crop species (1 seed per replicate) were placed uniformly above the treated soil. There were 5 replicates for each treatment, and all the cups were placed at the glasshouse and moistened to provide an optimum condition for seedling growth. The emergence, shoot dry weight, and root length of each bioassay species were recorded after two weeks of treatment, and the data were expressed in percentage with respect to control negative.

### Mode of action studies

#### Quantum yield determination

The study of quantum yield was referred to a modified method by Dayan and Zaccaro^34^. Compound **c** was dissolved in 1% acetone (v/v) to produce 5 ml of solution at 100, 300 and 500 mg/L in 5-cm diameter Petri dishes. Fully developed leaves of *O. verticillata* were punched into 8 mm diameter and dipped in the Petri dishes. Petri dish that contained 1% acetone solely acted as the control negative. An experiment was performed to determine the fluorescence produced by leaf discs at fixed time intervals of up to 24 h. All Petri dishes were placed in the growth chamber with constant temperature at 25 °C. The quantum yield was determined at the beginning 3 h and after 18 h of dark incubation and a final measurement after 6 h of light incubation using a handheld photosynthesis device CI-340 with an attached chlorophyll fluorescence meter (CID Bio-Science, Inc., Camas, WA, USA). The quantum yield is measured as percentage control with the following formula:

$${\text{Quantum}}\,{\text{Yield}}\,{\text{Percentage}}\,{\text{Control}}\,(\% ) = \frac{{{\text{Average}}\,{\text{quantum}}\,{\text{yield}}\,{\text{of}}\,{\text{treated}}\,{\text{leaf}}\,{\text{disc}}}}{{{\text{Average}}\,{\text{quantum}}\,{\text{yield}}\,{\text{of}}\,{\text{untreated}}\,{\text{leaf}}\,{\text{disc}}}} \times 100$$

#### Electrolyte leakage determination

The method mentioned by Dayan and Watson^33^ was modified to study the membrane permeability indicated by the electrolyte leakage of leaf discs samples. The leaf discs, which incubated in the dark for 3- and 18-h, followed by 6-h light incubation in the previous section of quantum yield, were reused to measure their electrical conductivity. Handheld electrical conductivity meter (Eutech Instruments) was used to measure the electrical conductivity of the buffer medium (EC1) before boiling. The content from each petri dish (leaf disc and buffer solution) was poured into a test tube and immersed in a hot water bath at a temperature of 95 °C for 20 min. After boiling, the buffer solution was left to cool before the second reading (EC2) was taken. The electrolyte leakage is expressed as percentage control using the following formula:

$${\text{Electrolyte}}\,{\text{leakage}} = \frac{{{\text{EC}}1}}{{{\text{EC}}2}}$$

$${\text{Electrolyte}}\,{\text{Leakage}}\,{\text{Percentage}}\,{\text{Control}}\,(\% ) = \frac{{{\text{Electrolyte}}\,{\text{leakage}}\,{\text{of}}\,{\text{treated}}\,{\text{leaf}}\,{\text{disc}}}}{{{\text{Electrolyte}}\,{\text{leakage}}\,{\text{of}}\,{\text{untreated}}\,{\text{leaf}}\,{\text{disc}}}} \times 100$$

#### Calcium antagonist determination

The assessment of calcium channel blockers was carried out following an earlier method described by Cho and Hong^36^ with some modifications. A preliminary test of sodium chloride, potassium chloride and calcium chloride solution on the seed germination of *E. indica* showed that the concentration of 50 mg/L of metal chloride solution did not have an adverse effect on seed germination *E. indica*^42^. Therefore, this concentration was chosen for examining the antagonistic effect of metal chloride on compound **c** and diuron. Germination tests were conducted with or without metal chloride solutions in the diuron and compound **c** at 125 mg/L, respectively, as described previously. After a week, the data of seed germination, shoot and root length of bioassay plants were documented and calculated in percentage respective to control negative, which contained water solely.

### Statistical analysis

#### Leaf disc discolouration test

Treatments were arranged in a completely randomized design with 5 replicates. No statistical analysis was carried out for the leaf disc discolouration test because the colour change of each replicate within the similar treatment was identical.

#### Germination test

Treatments were arranged in a complete randomized design with 5 replicates. All the percentage data for the germination test of **c** were fitted into the logistic function^43^, as below:

$${\text{Y}} = {\text{d}}/(1 + [{\text{x}}/{\text{x}}_{0} ]^{{\text{b}}} )$$

where Y is the percentage of germination, shoot length or root length, d is the coefficients corresponding to the upper asymptotes, x is the concentration of compound **c**, x_0_ is the concentration of compound **c**, which inhibits the germination, shoot length and root length by 50% for untreated seeds and b is the line gradient.

#### Pre- and post-emergence herbicidal test

Treatments were arranged as factorial in a complete randomized design with 5 replications where factor one is the type of phytotoxic compound, whereas factor two is the application rate. The percentages of data were checked for normality and homogeneity of variance before subjected to two-way ANOVA analysis. Tukey test was used to compare the mean between treatments at 5% of the significance level.

#### Mode of action determination

Each treatment was arranged in a completely randomized design with 6 replicates. All data for quantum yield and electrolyte leakage determination were tested for normality and homogeneity of variance before performing one-way ANOVA. For calcium antagonist determination, arcsine square root transformation was performed on data of diuron and compound **c** with or without the addition of metal chloride before subjected to one-way ANOVA. The Tukey test was used to compare the means between groups at 5% of the significance level.

## Supplementary Information

Supplementary Information.
