# Supplementary material for: Herbicidal properties of antihypertensive drugs: calcium channel blockers
Source: Sci Rep. 2021 Jul 9;11:14227. doi: 10.1038/s41598-021-93662-2 (PMC8270911; doi:10.1038/s41598-021-93662-2)
Supplement: Supplementary file 1 — Supplementary Information. [file 41598_2021_93662_MOESM1_ESM.docx]

**Supplementary Information**

**Herbicidal properties of antihypertensive drugs: Calcium channel blockers**

Hannan Safiyyah Tan Sian Hui Abdullah^1^, Poh Wai Chia^1^, Dzolkhifli Omar^2^, Tse Seng Chuah^3,*^

^1^ Faculty of Science and Marine Environment, Universiti Malaysia Terengganu, Kuala Nerus, Terengganu, Malaysia.

^2^ Faculty of Agriculture, Universiti Putra Malaysia, Serdang, Selangor, Malaysia.

^3^ Faculty of Plantation and Agrotechnology, Universiti Teknologi MARA, Arau, Perlis, Malaysia.

*Corresponding author’s email: chuahts@uitm.edu.my

**Table S1.** Visual assessment of leaf disc colour change.

| Score | Colour | Leave disc appearance | Phytotoxic effect |
| --- | --- | --- | --- |
| 1 | Green  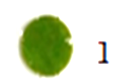 | The leaf disc surface is completely green | No |
| 2 | Green brownish  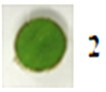 | 10 to 30% of the leaf disc surface is dark brown in colour | Weak |
| 3 | Brown greenish  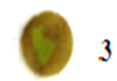 | 50 to 80% of the leaf disc surface is dark brown in colour | Moderate |
| 4 | Dark brown  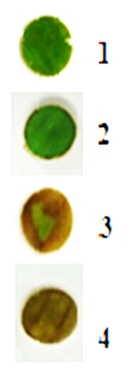 | More than 90% of the leaf disc surface, in aggregate, is dark brown | Strong |

##### ****Figure S1.** 1H NMR spectrum of **a**.**

**
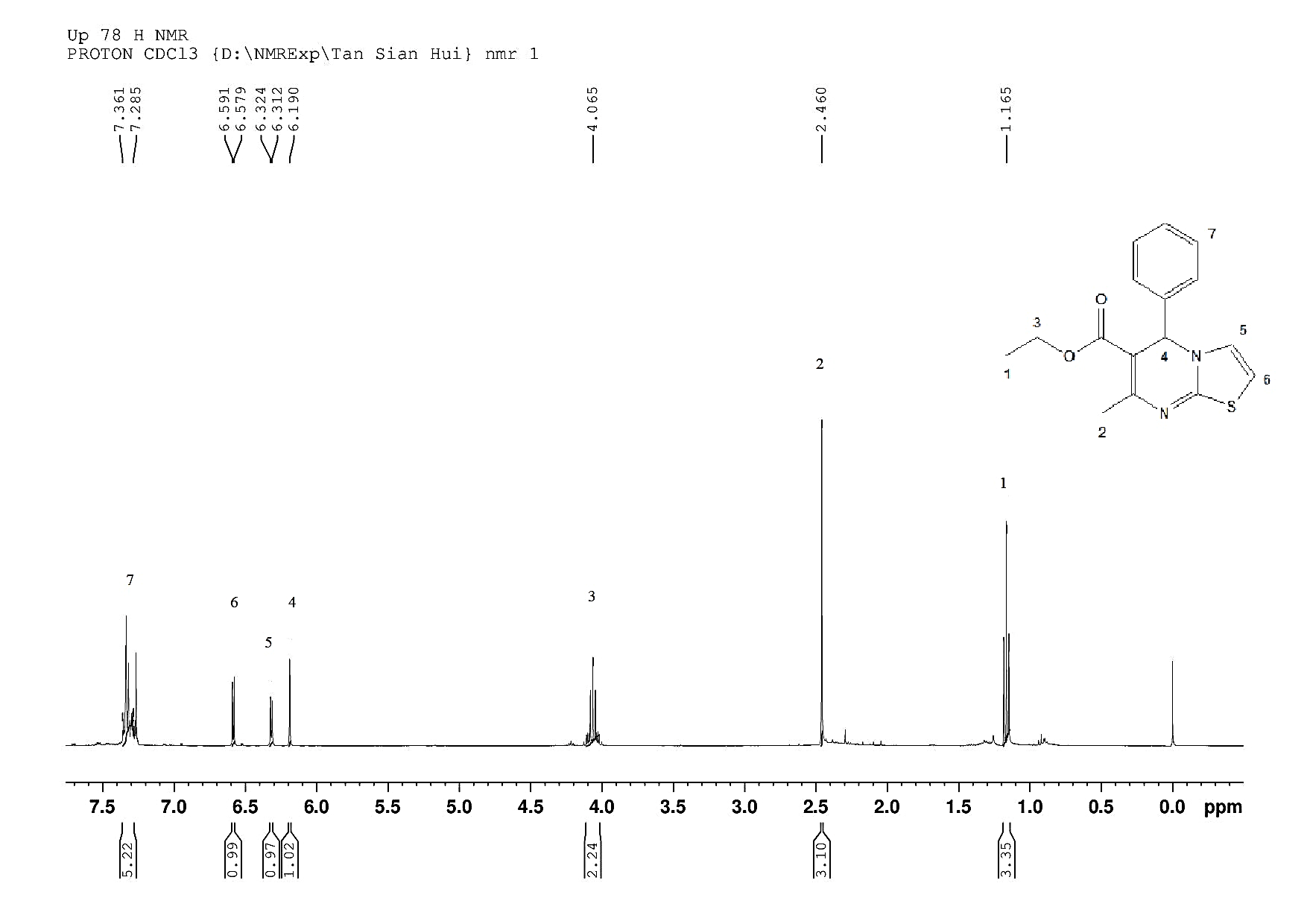
**

**Figure S2.**  1H NMR spectrum of **b**.

**
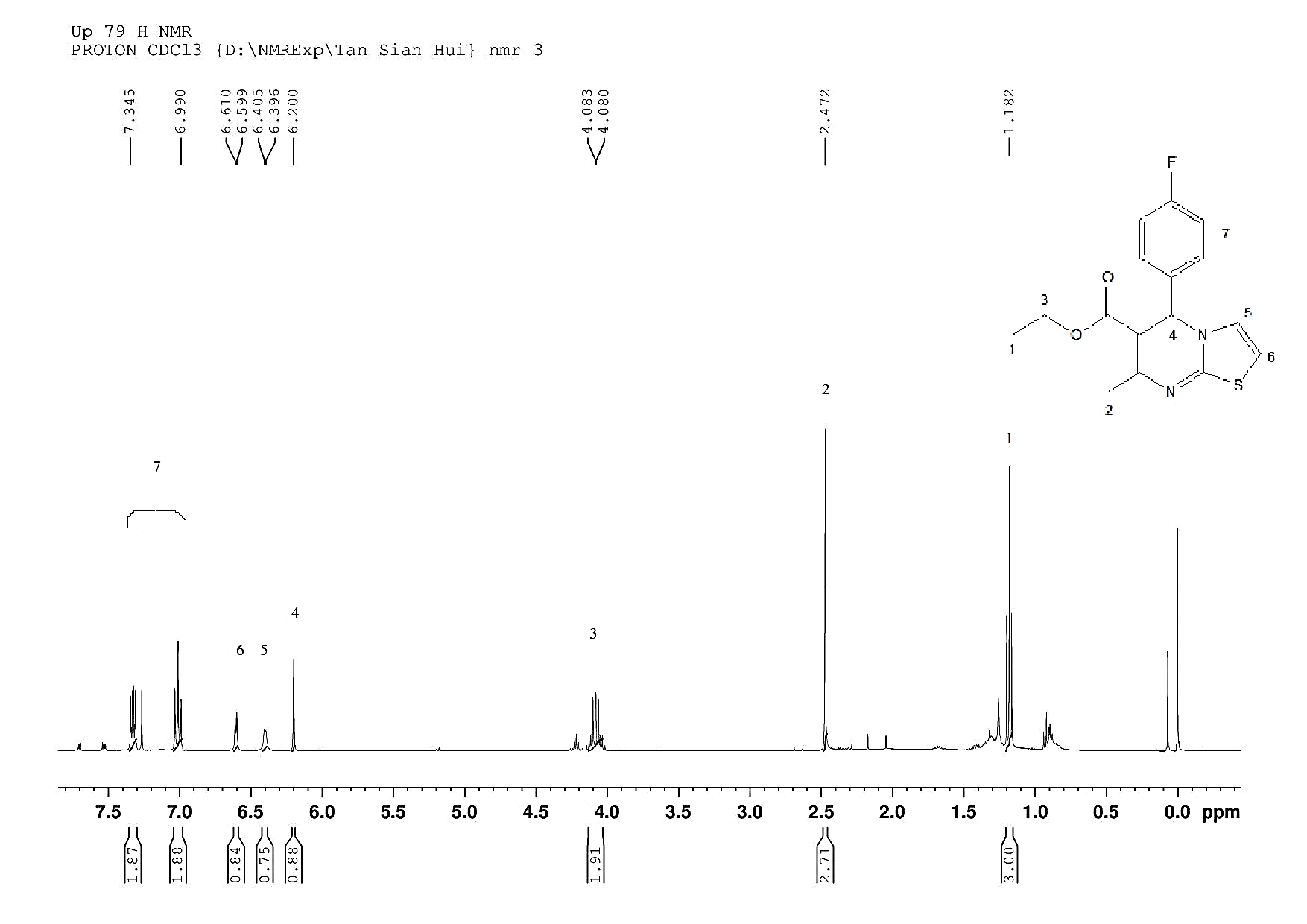
**

**Figure S3.**  1H NMR spectrum of **c**.


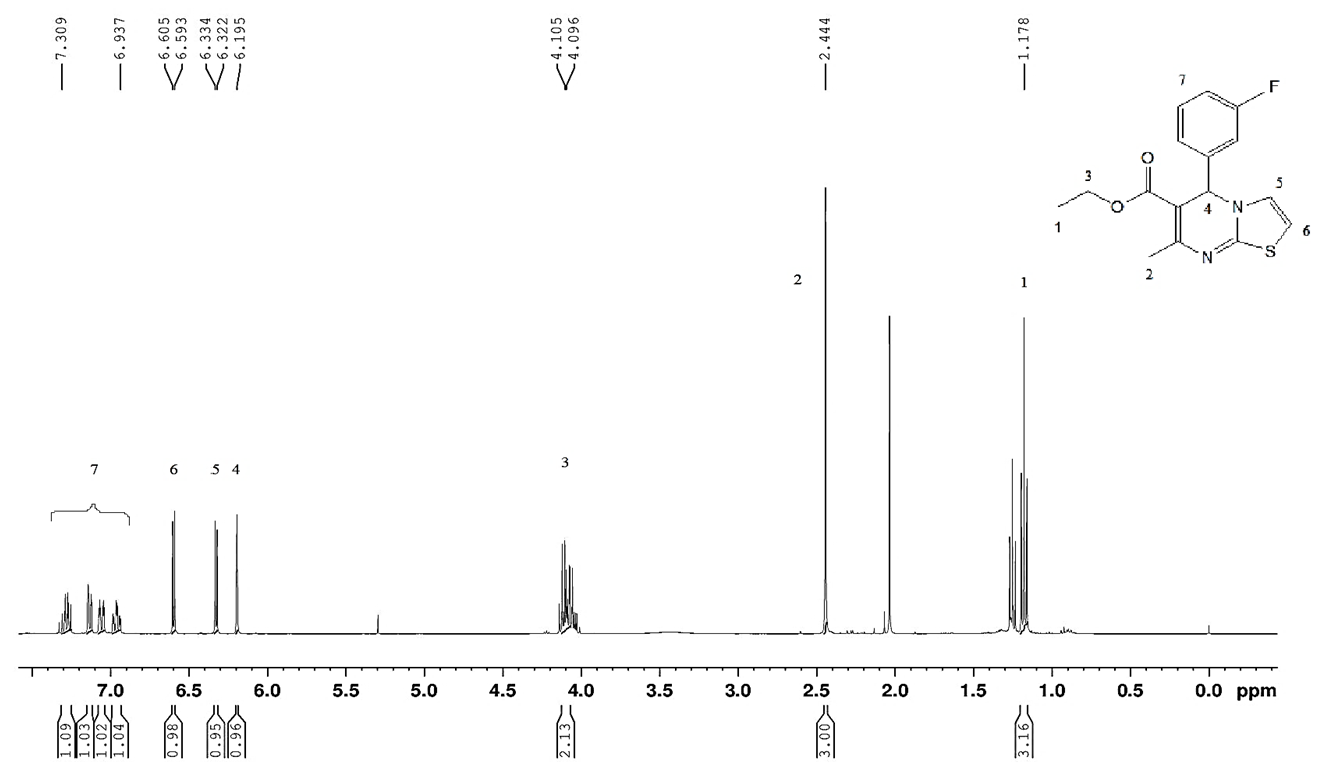


**Figure S4.**  1H NMR spectrum of **d**.

**
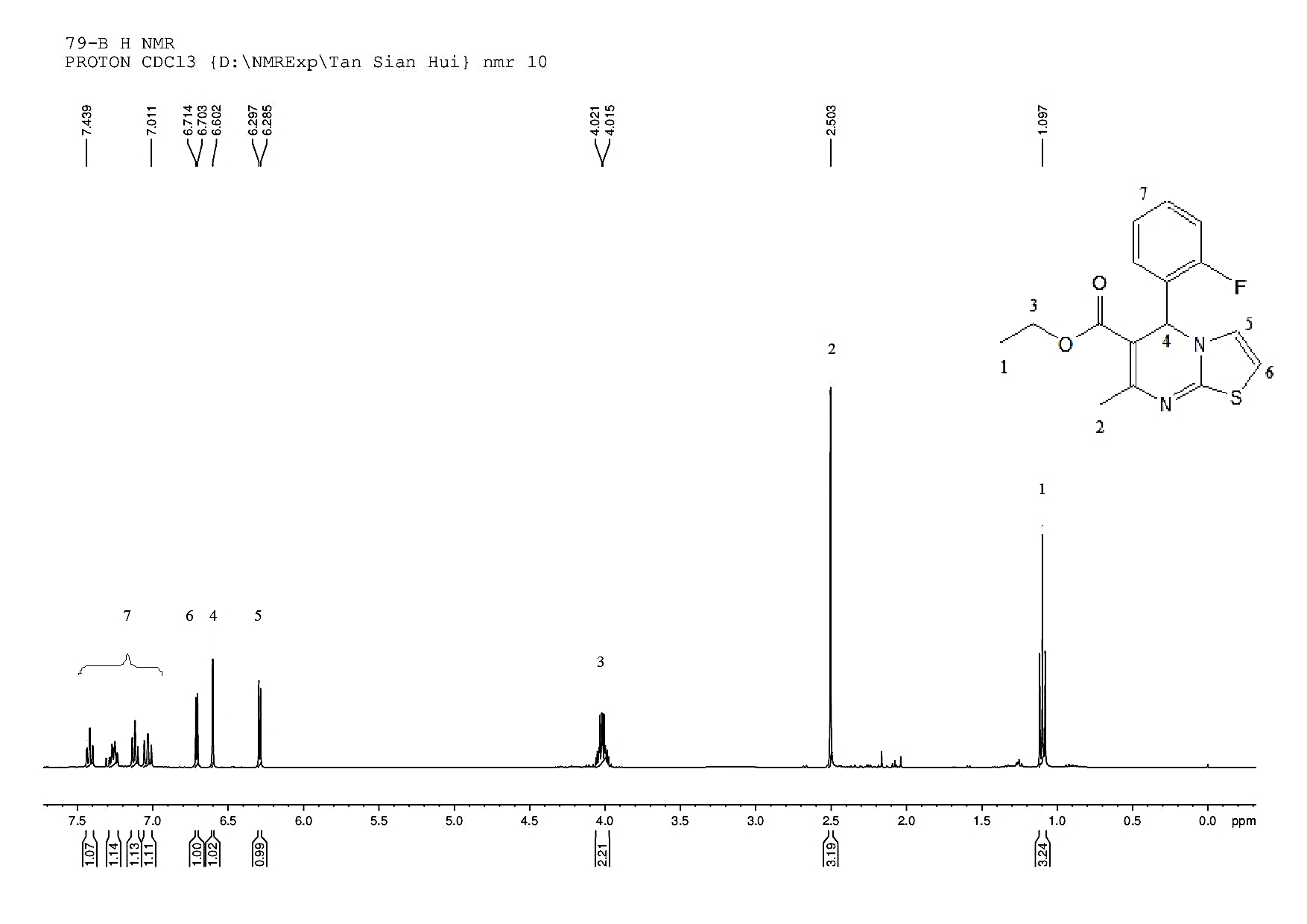
**

**Figure S5.**  1H NMR spectrum of **e**.

**
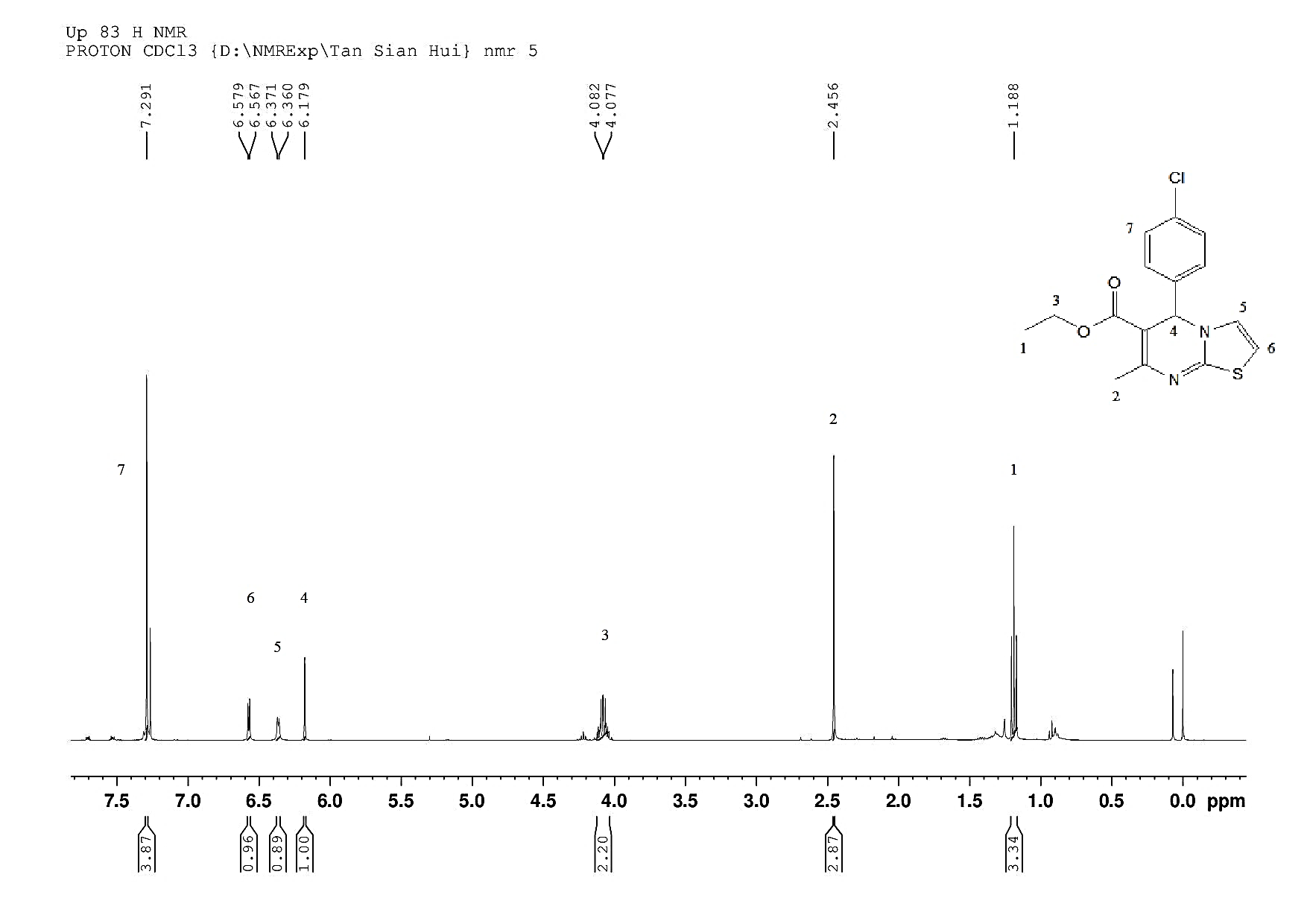
**

**Figure S6.**  1H NMR spectrum of **f**.

**
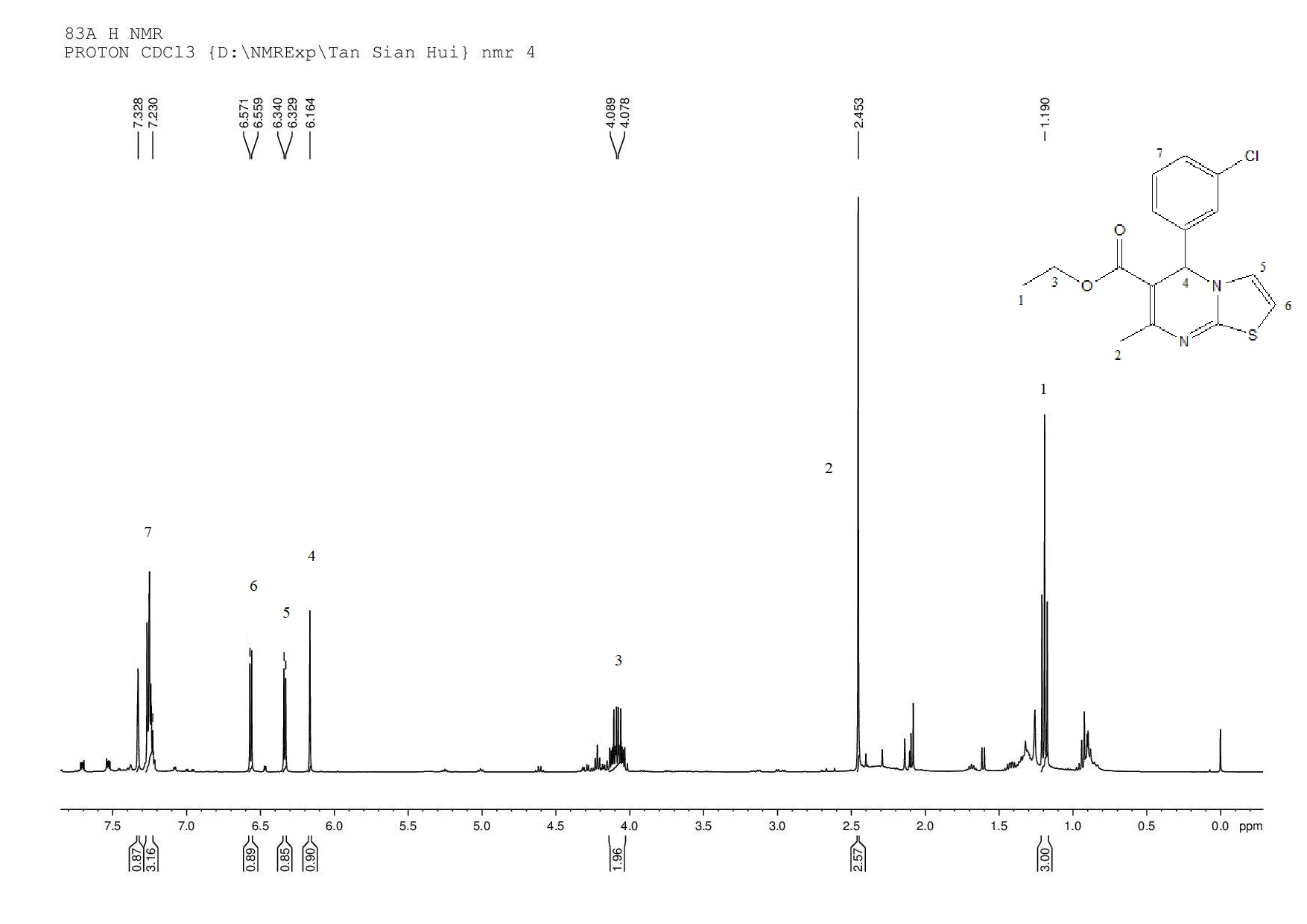
**

**Figure S7.** 1H NMR spectrum of **g**.

**
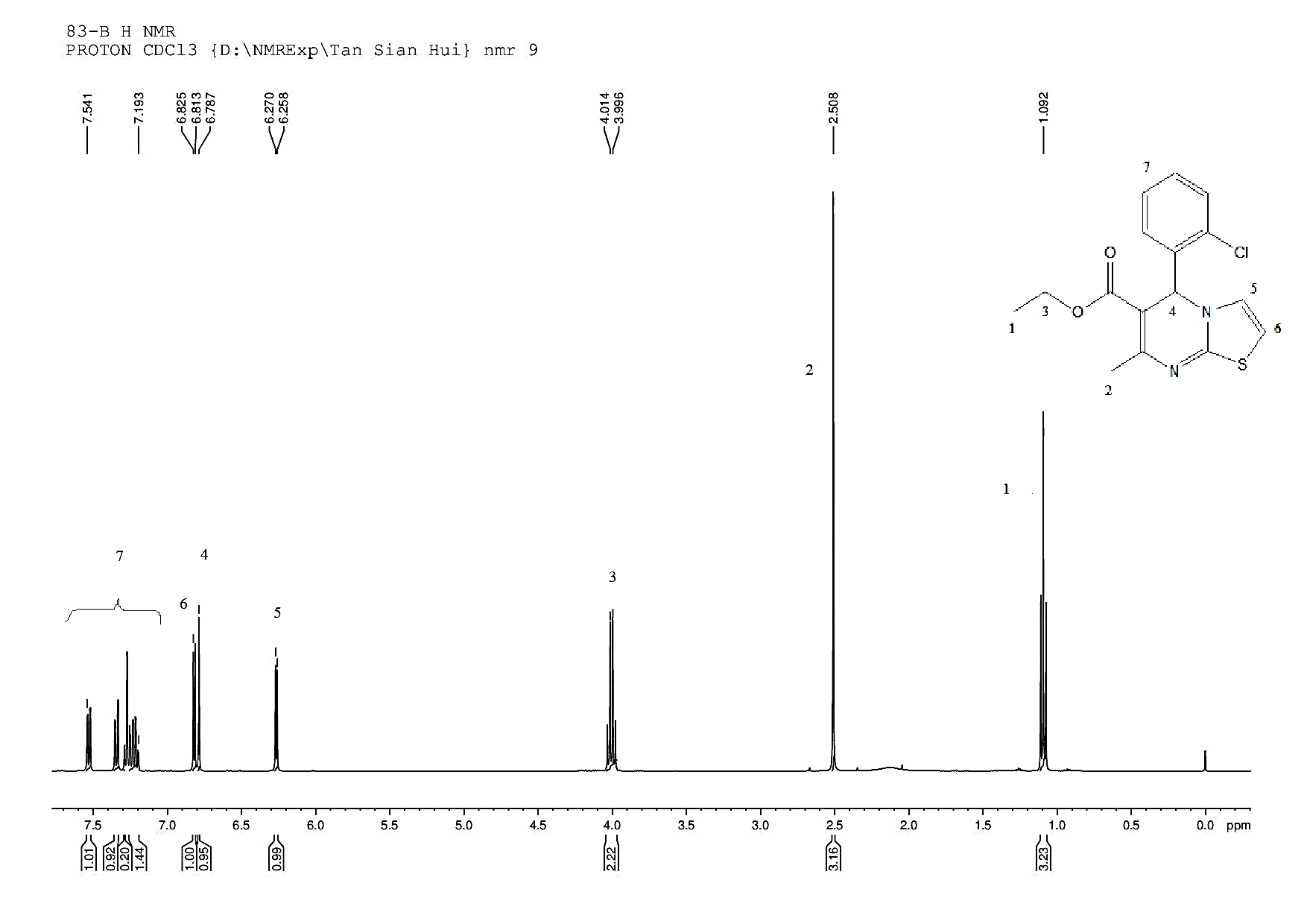
**

**Figure S8.**  1H NMR spectrum of **h**.

**
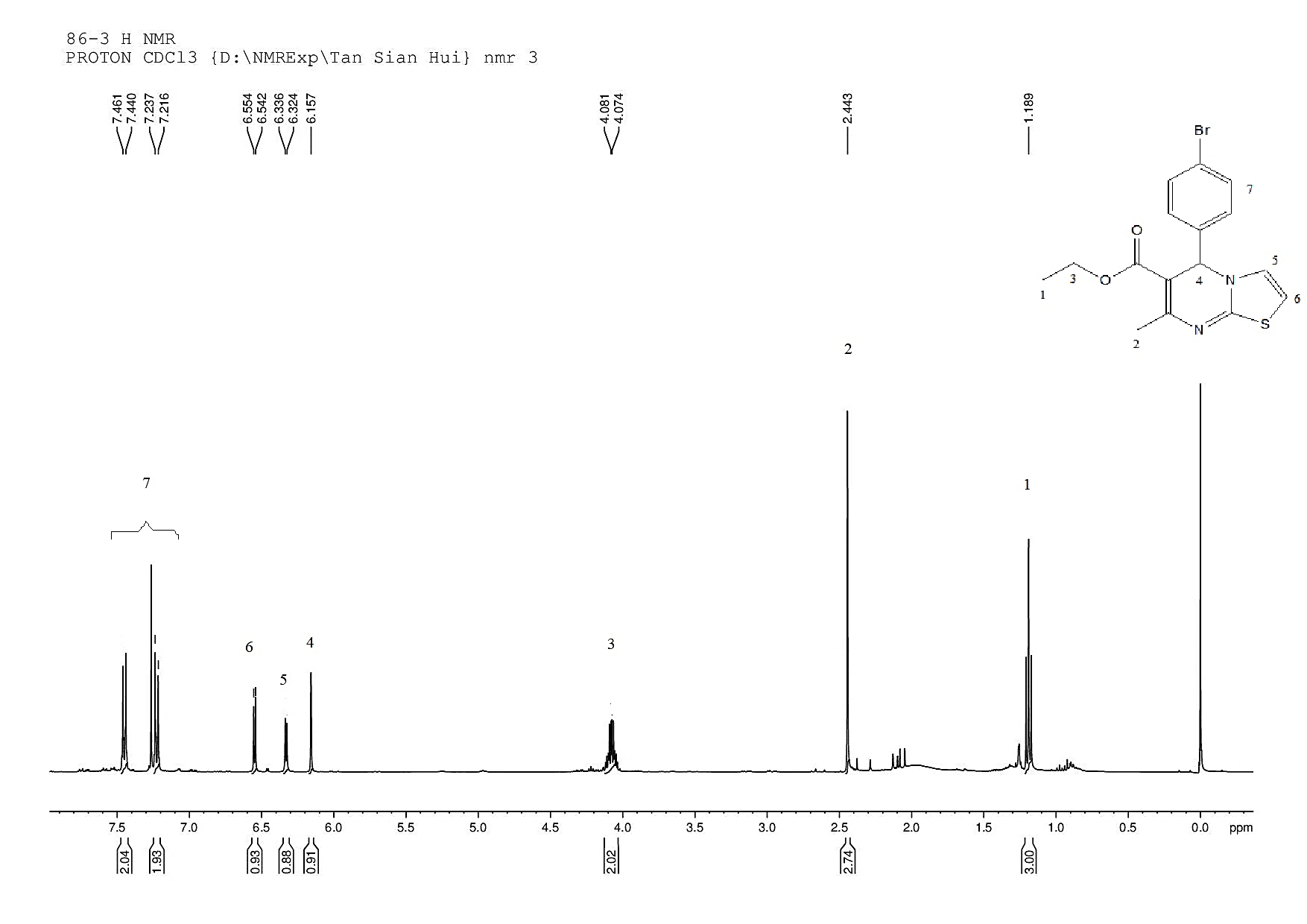
**

**Figure S9.**  1H NMR spectrum of i.

**
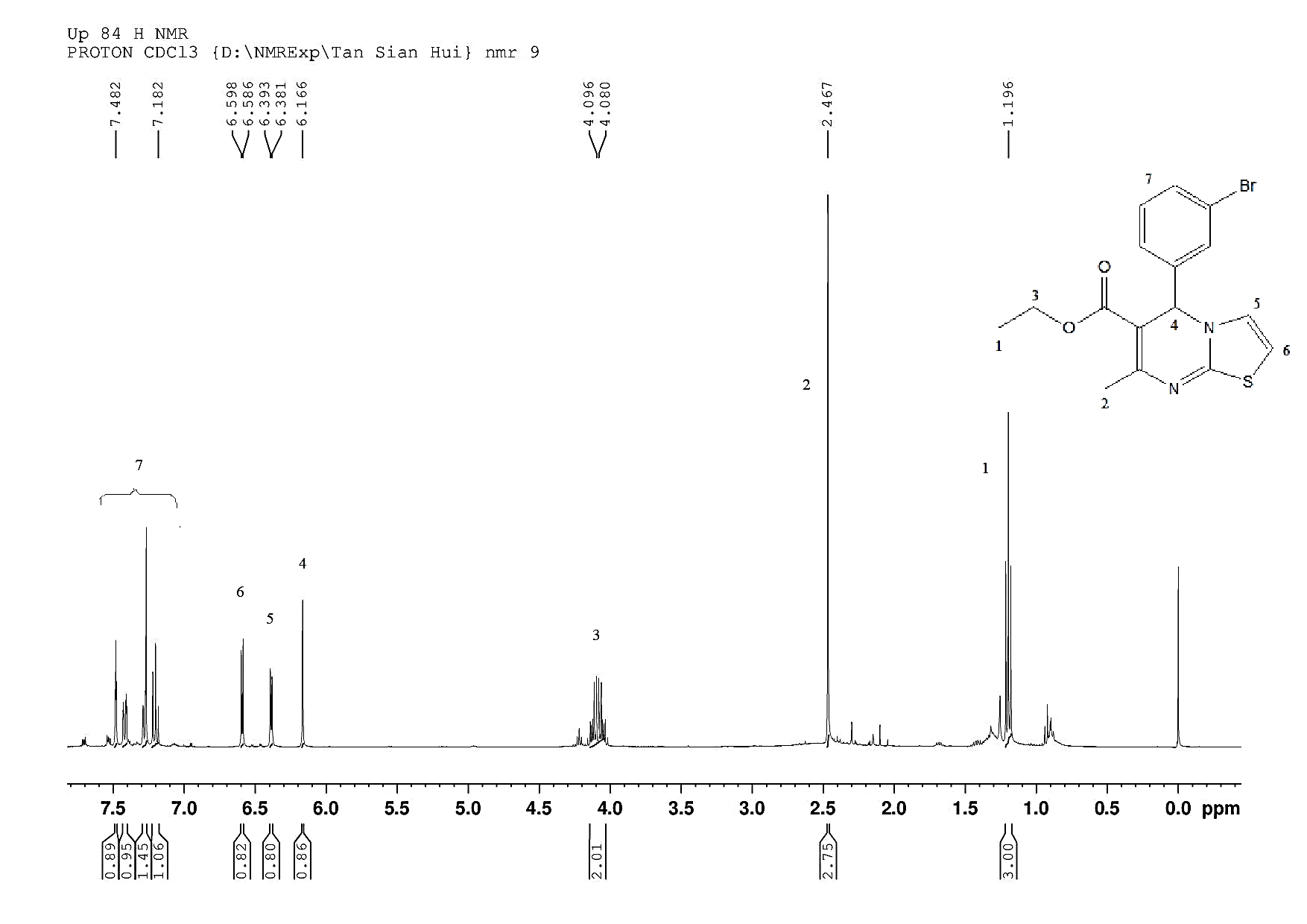
**

**Figure S10.**  1H NMR spectrum of **j**.

**
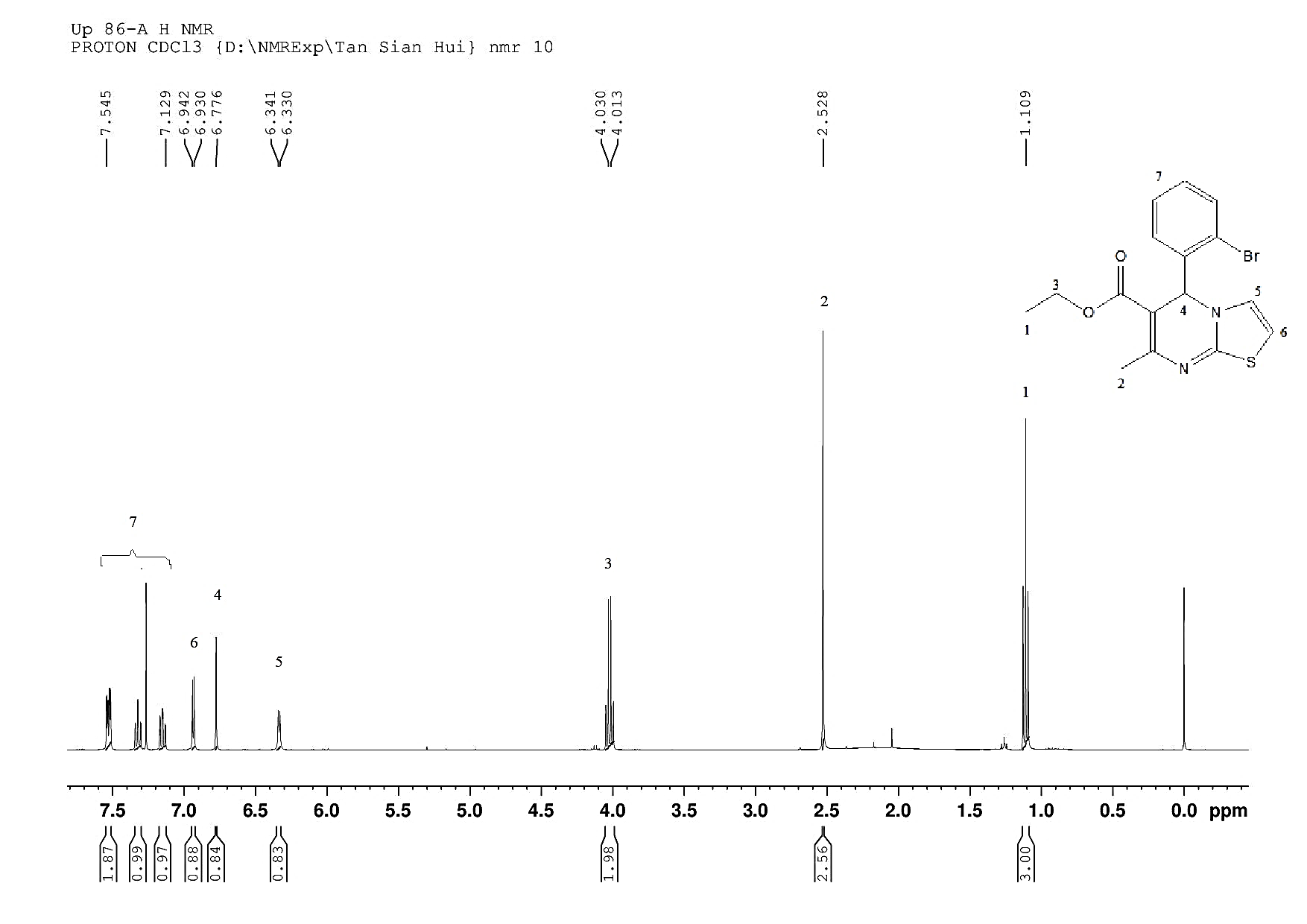
**
